# The incidence, risk factors, and mortality of preterm neonates: A prospective study from Jordan (2012-2013)

**DOI:** 10.4274/tjod.62582

**Published:** 2017-03-15

**Authors:** Nadin M. Abdel Razeq, Yousef S. Khader, Anwar M. Batieha

**Affiliations:** 1 The University of Jordan Faculty of Nursing, Department of Maternal and Child Health Nursing, Amman, Jordan; 2 Jordan University of Science and Technology, Faculty of Applied Medical Sciences, Department of Community Medicine and Public Health, Irbid, Jordan

**Keywords:** Preterm, infant, prematurity, obstetric, premature birth

## Abstract

**Objective:**

To explore the incidence of preterm delivery, maternal risk factors for having a preterm neonate, and preterm neonates’ mortality in Jordan.

**Materials and Methods:**

A cross-sectional population-based design was applied. Socio-demographic, perinatal, delivery risk factors, and survival information were gathered in pre- and post-hospital discharge interviews with 21075 women who gave birth to live neonates at ≥20 weeks of gestation in 18 hospitals in Jordan. Women were interviewed between 2012 and 2013. The sample was limited to singleton women who gave birth to live neonates. Women who gave birth to stillborn babies were excluded.

**Results:**

Preterm delivery incidence was 5.8%, of which 85% were in 32-36 gestational weeks. Male sex, primigravidity, hypertension, preeclampsia, and diabetes were significantly associated with an increased risk of preterm delivery. Women aged 20-35 years had the lowest risk of preterm delivery. Mother’s weight <50 kg, hospitalization at 24-34 gestational weeks, lack of antenatal care visits or <8 visits during pregnancy, a history of preterm delivery, and a history of stillbirth/neonatal death were associated with increased risks of preterm delivery. The neonatal mortality rate was 4/1000 live births among full-term and 123/1000 live births among preterm babies. Prematurity, congenital anomalies, and maternal diseases were the causes of 84% of preterm neonatal deaths.

**Conclusion:**

The mortality rate was considerably higher among preterm neonates than among term neonates; discrepancies between Jordan and other countries existed. Systematic prenatal risk assessment and quality postnatal health care improvements are required to improve the survival rates of preterm neonates.

## PRECIS:

The neonatal mortality rate was 30 times higher in preterm neonates than in term neonates, which indicates a survival gap between the two groups.

## INTRODUCTION

Prematurity presents a significant challenge to the global community due to the rapid increase in its incidence and its disproportionate contribution to increased infant mortality rates. In 2010, approximately 15 million babies were born preterm, and more than 1 million died due to complications during the first month of life^(1)^. Globally, among all neonatal deaths in 2013, 35% were caused by preterm birth complications alone^(2)^. Research that expands our understanding of the causes and risk factors of preterm birth and how to identify women and adolescents at risk is particularly needed to decrease the global neonatal mortality rate^(1)^. Without accurate, comprehensive background information describing the existing state of preterm neonatal births, risk factors, and national mortality, an international improvement in preterm neonatal care would be extremely challenging to achieve. Population-based studies reporting the outcomes of preterm birth using standardized mortality definitions are highly recommended in low- and middle resource settings^(3)^.

Jordan has a total fertility rate of 3.5 per woman, and a birth rate of 27 per 1000 people (2010-2012)^(4)^. Corresponding to the global picture, during 2013, half of the 4000 children in Jordan who died under the age of five years were neonates^(2)^. Jordan ranked 97^th^ globally in under-five mortality rates in 2012^(5)^. Despite a progressive decline in neonatal mortality rates between 1990 and 2013, the national neonatal mortality rate remains high, at 11 neonatal deaths per 1000 live births in 2013^(2)^. However, evidence regarding the incidence, geographic distribution, associated factors, and mortality risks of preterm births in Jordan is limited; these are mostly deduced from single settings or confined to data from limited geographic areas^(6,7,8,9,10,11,12,13,14)^. This study is part of a larger nation-wide study, conducted in 2012 and 2013, to examine perinatal mortality in Jordan^(15)^. The purpose of this paper was to report the incidence of preterm birth, its risk factors, and its contribution to neonatal mortality.

## MATERIALS AND METHODS

### Study design

This was a national, prospective, hospital-based study.

### Setting

The study was conducted at 18 maternity hospitals that were carefully selected based on criteria determined by the study’s technical committee, which consisted of representatives from the United Nations International Children’s Emergency Fund (UNICEF), the World Health Organization (WHO), and the Jordanian health sectors. These criteria reflected the diverse socioeconomic status of the participants and the quality levels of services provided to them. The hospital selection criteria also considered the workload of the hospitals in terms of the number of deliveries.

Accordingly, the 18 hospitals were distributed over the three regions of the country: seven hospitals in the middle, six in the north, and five in the south. The hospitals represented all health care delivery sectors in the three geographic regions of Jordan, including urban and rural areas. The number of births selected from each hospital was proportional to the number of births in each health sector and region. The Institutional Review Boards at the Ministry of Health and selected hospitals approved the study (approval number: 2012/035).

### Participants

All women who gave birth to dead or live neonates at 20 or more weeks of gestation in each of the selected hospitals were eligible for inclusion and were interviewed before discharge from the hospital. The sampling criteria and size of the larger study are described in detail elsewhere^(15)^. In the current analysis, the sample was limited to singleton women who gave birth to live neonates. Women who gave birth to stillborn babies were excluded. Written informed consent was obtained for each participant prior to commencing the interviews.

### Data source and measurements

A number of questionnaires and forms were developed, revised, and finalized by the study team to facilitate the gathering and recording of research data. The questionnaires had specific instructions, and the content was organized to increase clarity and enhance the accuracy of the obtained data. A team of 3-6 midwives/nurses, led by an obstetrician and a neonatologist or pediatrician, was assigned to collect data at each selected hospital. Qualified local trainers conducted a two-day training workshop for researchers working in each region. The study questionnaires and forms were pre-tested in the field on a sample of women similar to those who were actually included in the study.

The pre-discharge interview questionnaire contained information about the socio-demographic, maternal, and clinical characteristics of the women; information about pregnancy, labor, and delivery; relevant information about the neonates; and perinatal and neonatal deaths. Information was also gathered from the participating women’s medical records pre-discharge. Woman whose neonates died before discharge were asked further questions about the circumstances and causes of death.

For those women who did not experience perinatal or neonatal death before discharge, consent to follow-up with them postpartum was obtained, and their phone number was noted. The women who agreed to be contacted were called 30 days postpartum to participate in a phone interview; questions were asked about whether the neonate was alive, the health of the neonate, and details of visits to health facilities for both the mother and her neonate. If, through this screening call, it was discovered that a neonate had died, plans were made for a follow-up interview at the woman’s home for a “verbal autopsy”.

Neonatal gestational ages were recorded in the women’s medical records by practicing physicians. The gestational age was based on both ultrasound and the last menstrual period (the interval between the first day of the mother’s last normal menstrual period and the date of delivery of the fetus or newborn). A preterm neonate was defined as a neonate that was born before 37 completed weeks of pregnancy. Based on the gestational age, preterm babies were further classified as born between 32-36 weeks of gestation and born at <32 weeks. A birth weight less than 2500 grams (5 pounds, 8 ounces) was considered as low birth weight and a birth weight of 2500 grams and above was considered as normal weight. The neonatal mortality rate was defined as the number of deaths during the first 28 completed days of life per 1000 live births that occurred during the study period. Neonatal deaths were subdivided according to the time of death into early neonatal deaths at 0-6 days after live birth and late neonatal deaths at 7-27 days after live birth. Both were expressed as per 1000 live births.

The hospital diagnosis of the causes of neonatal deaths was based on standard obstetric and neonatal guidelines. The deaths were classified according to the National Institute for Heath and Care Excellence (NICE) system, a standard classification system that is based on the modified Wigglesworth classification(16), together with information on the calculation of birth weight in relation to gestational length. The NICE classification program classifies neonatal death into one of a range of specific, mutually exclusive, cause-of-death subgroups. The order of the subgroups is strictly hierarchical.

### Statistical Analysis

Data were described using means (standard deviation) for continuous variables, and frequencies and percentages were used for categorical variables. The differences between proportions were tested using chi-square tests. Risk factors were analyzed using generalized, linear-mixed multilevel models, and traditional logistic regression analysis was used to measure the hierarchical complexity of predictor variables. The Akaike information criterion (AIC) and the Bayesian information criterion were used to select and compare models based on the -2 log likelihood. Based on the information criteria, we preferred the final binary logistic regression model predicting prematurity over the final generalized linear mixed model with one random intercept, because it had smaller AIC values. Based on a likelihood ratio test, the binary logistic regression (no random effect) was still preferred. The variables were included in the model systematically, and those with p<0.10 in the univariate analysis were included in the model. The possible associated factors were examined for evidence of multicollinearity, which was reflected by either the changes in the direction of effect between the univariate and multivariable analysis or implausible standard errors for a particular variable. A p-value of <0.05 was considered statistically significant. Traditional and multilevel analyses were performed using IBM SPSS 20 (SPSS Inc., Chicago, IL, USA).

## RESULTS

### Participants’ characteristics

During the study period, 21 075 women gave birth in the selected hospitals. Approximately one third of deliveries (34.2%) occurred in the northern region, 55.0% in the middle region, and 10.8% in the southern region. About 30.5% of women gave birth in private hospitals, 46.9% in public hospitals, 18.7% in military hospitals, and 3.8% in teaching hospitals. Almost all women (99%) sought antennal care, with 70.1% seeking antennal care >8 times. Less than one-third (32%) of women had an education level that was lower than high school, and 41.5% had an income of >350 Jordanian dinar (JD) per month (1 $= 0.71 JD). About 8.0% of women had a history of preterm or low-birth-weight delivery, 3.2% were smokers, 1.2% had preeclampsia, 1.2% had gestational diabetes, 0.5% had pre-gestational diabetes, and 4.8% had pre-gestational high blood pressure.

### Incidence of preterm birth

Among the participants, 5.8% delivered before 37 completed weeks of gestation. The majority of preterm deliveries (85%) occurred between 32 and 36 weeks, and 15% reached <32 weeks of gestation. The preterm birth rate, according to the socio-demographic and clinical characteristics of the mothers and their babies, is shown in Table 1. The preterm birth rate was the lowest among women aged 20-35 years (7.8% for women aged <20 years, 5.4% for women aged 20-35 years, and 7.7% for women aged >35 years). The rate was extremely high among women who did not use antenatal care services (13.8%). The rate was also higher among women who had a history of preterm/low-birth-weight delivery (14.4%), hypertension (12.6%), preeclampsia (24.7%), gestational diabetes (10.3%), pre-gestational diabetes (25.7%), and women who were hospitalized between 24 and 34 gestational weeks. The rate of cesarean section births (46.4%), both planned and emergency, among the preterm neonates was almost equal to that by vaginal delivery (50.7%).

The main characteristics of the full-term and preterm deliveries are shown in Table 2. Presentation at delivery was cephalic in 95.4% of full-term deliveries and 86.9% of preterm deliveries. About 27% of full-term deliveries and 46.4% of preterm deliveries were performed via cesarean section. Neonatal resuscitation was necessary for 9.6% of full-term babies and 29.2% of preterm babies. Preterm babies were more likely to have poor Apgar scores at 1 and 5 minutes compared with full-term babies.

### Risk factors of preterm delivery

In multivariable analysis (Table 3), male sex [odds ratio (OR)=1.2], primigravid (OR=1.6), hypertension (OR=1.5), preeclampsia (OR=3.1), and diabetes (OR=1.6) were significantly associated with an increased risk of preterm delivery. Women aged between 20 and 35 years had the lowest risk of giving birth to a preterm neonate compared with older or younger women. A mother’s weight <50 kg, hospitalization at 24-34 gestational weeks, no antenatal care visits or <8 visits during pregnancy, and a history of preterm or low-birth-weight delivery or stillbirth/neonatal death were all associated with an increased risk of preterm delivery.

### Rates and causes of neonatal mortality among preterm and full-term babies

The neonatal mortality rate was four per 1000 live births among full-term (early neonatal death=three per 1000 live births, and late neonatal death=one per 1000 live births). The neonatal mortality rate was 123 per 1000 live births among preterm babies (early neonatal death=99 per 1000 live births, and late neonatal death=24 per 1000 live births). Among normal birth weight babies, the neonatal mortality rate was 3 per 1000 live births for full-term and 22 per 1000 live births for preterm babies. Among low-birth-weight babies, the rate was 32 per 1000 live births for full-term, and 116 per 1000 live births for preterm babies. The causes of death for full-term and preterm babies are shown in Table 4.

## DISCUSSION

The incidence of preterm births in the current study was 5.8%, comparable to that reported in developed countries such as Finland, Ireland, and Sweden(17). The preterm birth rate in this study compares positively with that observed in the United States and in most low- and middle-income countries^(17,18,19,20)^. The relatively lower incidence rate in this study may be due to the larger number of participants aged 20-35 years and educated participants. Evidence from previous studies indicated that young (<20 years) and advanced (≥40 years) maternal ages are strong risk factors of preterm births^(9,21,22)^. Similarly, increased risks of mothers having a preterm birth were associated with low or no education levels^(21)^.

The incidence rate of preterm birth noted in this study was also lower than that reported in earlier studies in Jordan^(7,9,10)^. The proportion of preterm births, as reported by single-setting studies conducted in Jordan, ranged between 10.7%^(7)^ and 12.8%^(10)^ out of the total births in each setting, and the highest mortality in the neonatal intensive care unit was among preterm and low-birth-weight admissions^(12)^. The discrepancy in the incidence of preterm births between the current study and previous studies in Jordan could be due to the small samples and geographic areas studied previously. The lower preterm birth rate in our study could indicate the positive progress that has been made recently in the quality of maternal-fetal health care in Jordan. The high number of women in the current study who attended antenatal clinics supports this supposition.

However, the high attendance rate of antenatal care in this study did not reflect positively on the preterm neonatal mortality rate. The mortality rate of 123/1000 was relatively high. The neonatal mortality rate was 30 times higher among preterm neonates than among full-term neonates, indicating a survival gap between the groups; this disparity may be perceived as an urgent call for the systematic improvement of postnatal and neonatal intensive health care in Jordan. The best intervention for prevention of spontaneous preterm birth in women with risk factors is still unclear^(23)^. However, simple cost-effective and research-supported interventions are available to reduce deaths among premature babies; for example, the promotion of early and exclusive breastfeeding, handwashing, and innovative skin-to-skin care^(24,25)^. The prevention of hypothermia and management of respiratory distress syndrome, neonatal pneumonia, sepsis, and hyperbilirubinemia are evidence-based interventions that can greatly increase the survival of small and sick neonates^(24)^.

Globally, 4 out of 5 newborn deaths result from three preventable and treatable conditions, primarily prematurity^(25,26)^. Prematurity alone was the direct cause of almost 50% of neonatal deaths in the current study, followed by congenital anomalies and maternal medical conditions. Prematurity is often complicated by infections and respiratory complications, which commonly leads to the death of preterm infants^(19,27)^. These complications can be prevented and treated by skilful and high-quality postnatal care of preterm neonates, especially during the first week of life. Our findings indicate that preterm neonates were four times more likely to die postnatally during the first week of life, compared with later times after birth. Nevertheless, high-quality antenatal screening and care are still key components in efforts to identify preterm birth risk factors early, prevent preterm births, and reduce infant mortality.

This research showed that the incidence of preterm birth was significantly reduced when mothers received health care in antenatal clinics during pregnancy. The more antenatal visits mothers attended during pregnancy, the lower was the risk of preterm births. Previous studies have shown that preterm births were significantly more common among women who had no or only occasional visits to antenatal care^(27,28,29,30)^. In this study, the risk of preterm birth was almost four times greater in women who did not attend antenatal care, a risk ratio that is consistent with a study in Thailand^(29)^.

Globally, the proportion of women receiving antenatal care at least once during pregnancy was 83% between 2007 and 2014. However, only 64% of pregnant women attended the WHO-recommended minimum of four or more antenatal care visits^(31)^. Correspondingly, in this study, almost all of the women had received antenatal care at least once. However, nationally, this is not sufficient, because more than one quarter of the sample received less than the national goal of a minimum of eight visits; this indicates a need to improve women’s access to and compliance with antenatal health services. These results may influence health policies in Jordan and globally.

The identification of warning signs during pregnancy is an important goal of antenatal care^(31)^. Preeclampsia, diabetes, and hypertension, whether pre-existing or gestational, are maternal medical conditions that commonly predict preterm birth^(20,32)^, a finding that is similar to those of this study. For women with preeclampsia, the risk of preterm delivery was three times greater than it was for women who were not affected. This highlights that screening and medical management during antenatal care are clinically important to decrease the risk of preterm birth. Madan et al.^(33)^ found that the risk of preterm birth was augmented for obese and overweight mothers if they experienced one or more of the conditions listed above. Their conclusion highlights the importance of including weight indices in the assessment of preterm birth risk factors. The evidence suggests an increase in the likelihood of preterm birth when body mass index (BMI) decreases below or increases above normal^(29,33)^. Due to too many missing data, the researchers were unable to include BMI in the statistical analysis of their study; however, their results provide additional evidence of the role of underweight mothers on the increased risk of preterm birth.

Multiparous women with a history of preterm birth are also at risk for further preterm birth^(21,29,33)^. In the current study, the likelihood of having a preterm birth at least doubled when there was a history of preterm or low-birth-weight delivery in previous pregnancies; a rate similar to that reported in a Canadian study^(21)^. Likewise, primigravida was associated with a 1.6 increase in the likelihood of giving birth to a preterm neonate, which is approximate to the findings of similar studies^(21,29)^. The rate of caesarian section births, both planned and emergency, among the preterm neonates was remarkably high (46.4%) and is worth further investigation.

Maternal hospitalization during 24-34 weeks of gestation was associated with a very high likelihood of preterm delivery. This finding is understandable because early maternal hospitalization during pregnancy indicates the existence of maternal or fetal health problems or early identification of a potential problem. The reasons for hospitalization and types of care provided need more investigation because these factors could lead to a better understanding of preterm birth risk factors and hence prevent prematurity complications and preterm neonate mortality. Interventional studies that incorporate the results of this study in preterm delivery risk assessment in maternal and child health centers are also encouraged.

### Study Limitations

Although this study had several strengths compared with previous studies, it also had several limitations. Among the strengths of the current research were that it was nation-wide, covered a wide geographic area, and included information from all birth records in all sectors and types of hospital settings. It was not feasible, however, to include information on deliveries that occurred outside formal birth settings, such as private homes, which do occur in Jordan, albeit rarely. Although not all of the results of this study can be generalized to other countries, efforts were made to follow the WHO’s recommended definitions of prematurity and international standards of reporting mortality to allow for international comparisons and to enhance generalizing the data. In addition, this study was conducted over a specific period; therefore, replicating this study is highly recommended in future years to compare trends over time and identify changes in preterm birth estimates. Moreover, a longitudinal cohort study is strongly encouraged to follow preterm neonates because it would be highly beneficial to identify the long-term outcomes of preterm births, and the health needs of babies who survive prematurity.

## CONCLUSION

Addressing the major risks associated with the incidence and the mortality of preterm neonates is a priority to reduce the global burden of preterm birth, along with identifying areas that are crucial to improve the health care systems across countries. Regarding risk factors, the limited research carried out in Jordan shows that the rate of preterm and low-birth-weight infants was highest for males and first-born neonates^(10)^ among teenage women^(8,13)^ and women aged 35 years or above^(9,10^), as well as for women in consanguineous marriages^(11)^. By adding relevant information from Jordan, this study has contributed evidence to international comparison tables, and to the national as well as the international picture about prematurity.

## Figures and Tables

**Table 1:**
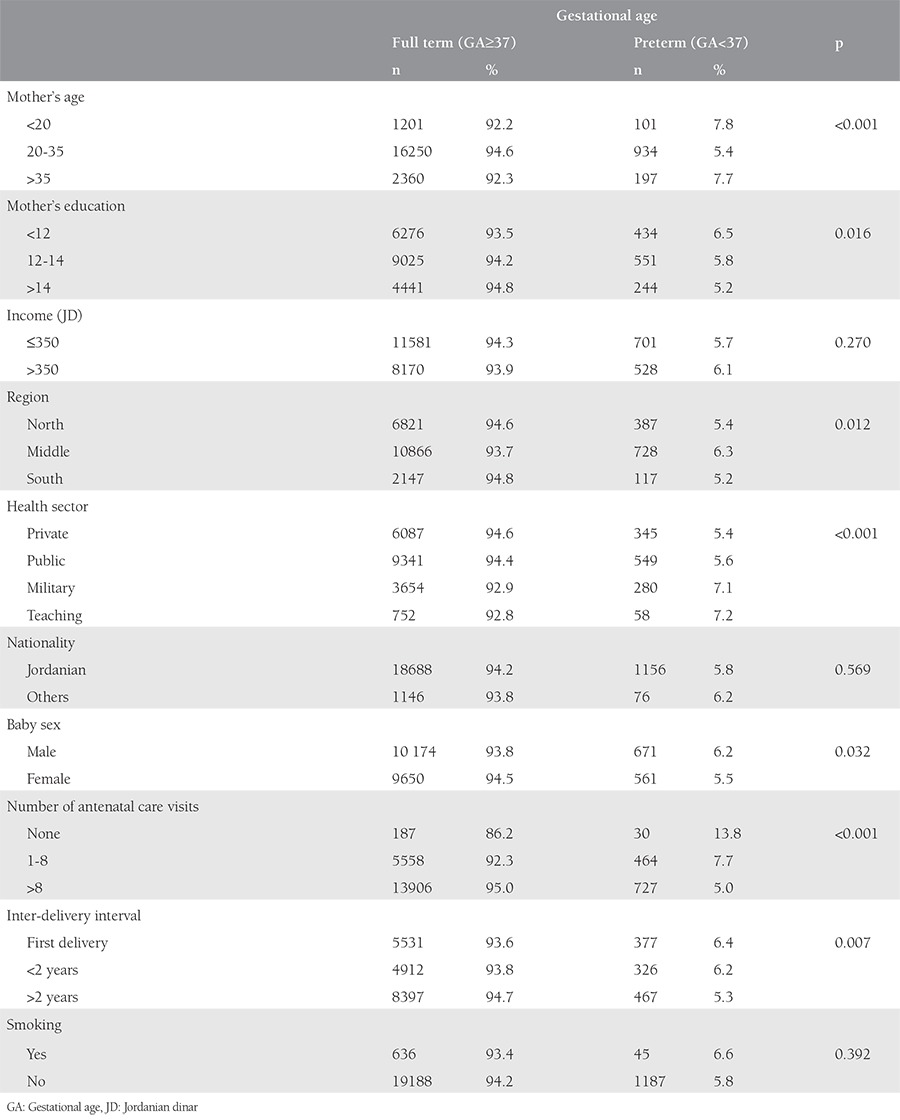
The rate of preterm birth according to socio-demographic and clinical characteristics of mothers and their babies

**Table 2:**
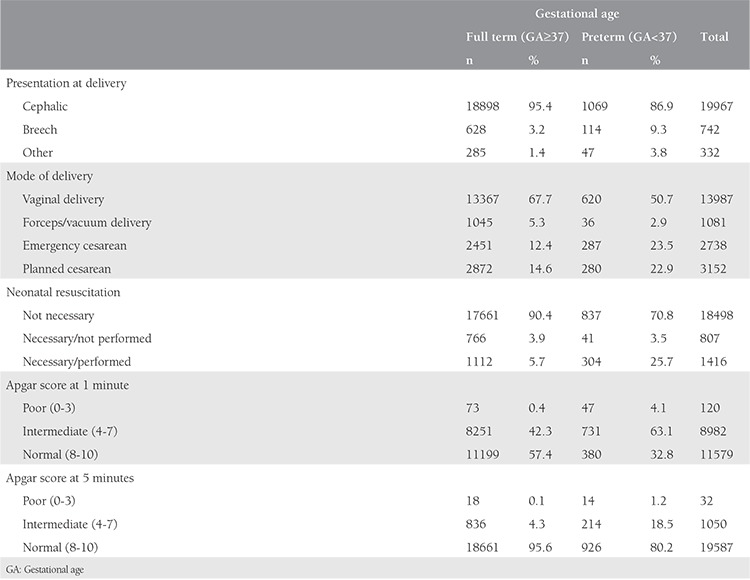
The main characteristics of full and preterm deliveries

**Table 3:**
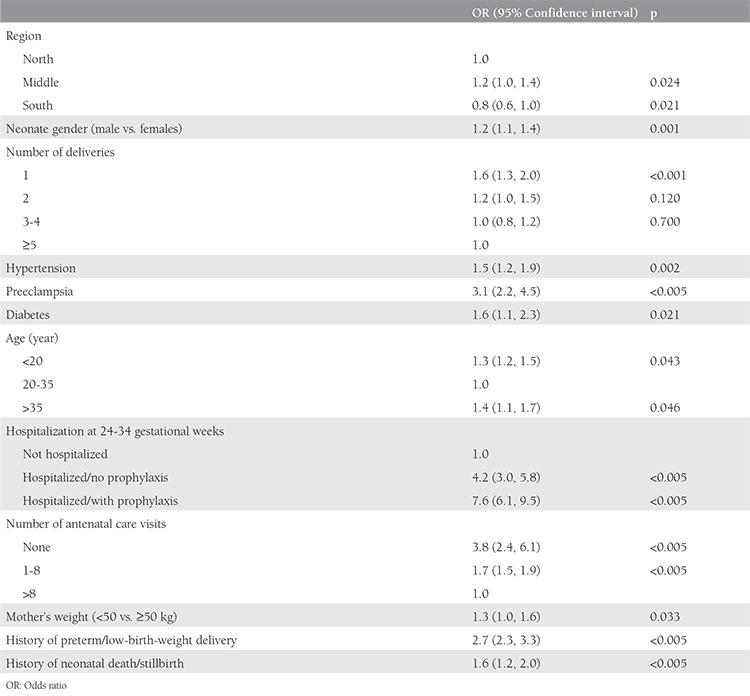
Multivariable analysis of factors associated with preterm delivery

**Table 4:**
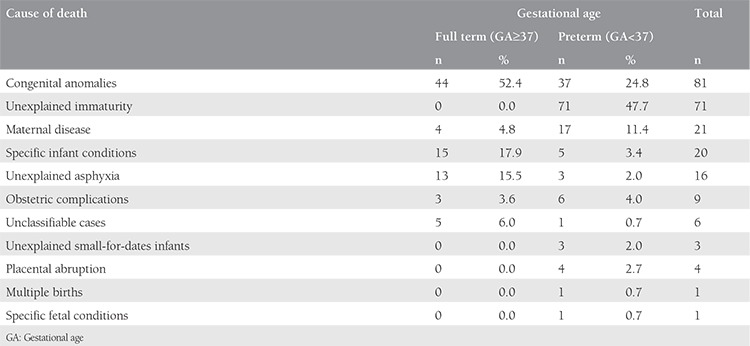
The leading causes of neonatal deaths among full term and preterm babies, based on the National Institute for Heath and Care Excellence classification
